# Associations of dietary antioxidant micronutrients with the prevalence of obesity in adults

**DOI:** 10.3389/fnut.2023.1098761

**Published:** 2023-03-13

**Authors:** Yazhu Yang, Haifeng Xu, Yi Zhang, Lin Chen, Chengzi Tian, Bihui Huang, Youpeng Chen, Lin Ma

**Affiliations:** ^1^Department of Reproductive Medicine Center, The Seventh Affiliated Hospital of Sun Yat-sen University, Shenzhen, China; ^2^Department of Infectious Diseases, The Seventh Affiliated Hospital of Sun Yat-sen University, Shenzhen, China; ^3^Scientific Research Center, The Seventh Affiliated Hospital of Sun Yat-sen University, Shenzhen, China

**Keywords:** antioxidant micronutrients, obesity, WQS regression, RCS regression, NHANES

## Abstract

**Background:**

Antioxidant micronutrients have a therapeutic potential for clinical treatment of obesity. NO research, however, has examined the connection between the complex level of dietary antioxidants and obesity.

**Materials and methods:**

We mainly aimed to investigate the relationship between a combination of antioxidants and obesity using the database of the national health and nutrition examination survey (NHANES). This cross-sectional study contains a survey of 41,021 people (≥18 years) in total ranging from 2005 to 2018. Multivariate logistic and weighted quantile sum (WQS) regression were performed to investigate the associations between these antioxidants, both individually and collectively, and the prevalence of obesity. The restricted cubic spline (RCS) regression was also utilized to analyze the linearity of these associations.

**Results:**

According to multivariate logistic models, we found that the levels of most antioxidants in the highest quartile were independently related to a lower prevalence of obesity, while a reverse result was observed in selenium (*p* for trend <0.05). The WQS index revealed that a total of the 11 antioxidants is negatively related to the prevalence of obesity and abdominal obesity (all *p*<0.001), and iron/vitamin C have the greatest weight in the negative associations between antioxidant complex and obesity, as well as abdominal obesity. In addition, the RCS regression showed that retinol, vitamin A, α-carotene, β-carotene, β-cryptoxanthin, vitamin C, iron, and copper all had a non-linear association with obesity. Threshold effect analysis demonstrated that the inflection points of retinol, vitamin A, α-carotene, β-carotene, β-cryptoxanthin, vitamin C, iron, and cooper were 235.57, 374.81, 58.89, 891.44, 30.70, 43,410.00, 11,240.00, and 990.00 μg/day, respectively.

**Conclusion:**

Our study found that a high level of a complex of 11 dietary antioxidants is related to a lower prevalence of obesity and abdominal obesity, among this inverse associations iron and vitamin C have the greatest weight.

## 1. Introduction

Obesity (1), a chronic metabolic condition, is classified into two types: abdominal obesity and non-abdominal obesity. Abdominal obesity, which is resulted from an excess of buildup of fat caused by the imbalance between the body’s caloric intake and consumption, is the result of the interplay of genetics, environmental variables such as poor food, long-term inactive and insufficient activity, and other factors (2–5). According to a research based on NHANES population, the prevalence of obesity among U.S. adults was 43.4% (6). Obesity is considered a global pandemic, with health hazards affecting practically everyone, particularly abdominal obesity, that is more dangerous than non-abdominal obesity. Obesity (7) also raises the risk of cardiovascular illnesses including atherosclerosis, venous thrombosis, and hypertension, as well as diabetes, cancer, and even all-cause mortality. According to a WHO research in 2018 (8), at least 2.8 million individuals worldwide died of overweight or obesity each year. This is why controlling and managing obesity is critical.

The removal of reactive oxygen species (ROS) that harm an organism is a process known as anti-oxidation. To attain the objective of antioxidation, people can supplement with antioxidants (including vitamins such as vitamins A, C, and E, carotenoids such as α-carotene and β-carotene), or supplement with a suitable quantity of proteins and trace elements such as iron, zinc, selenium, copper, retinol, and so on (9–11). These elements (12–14) are also currently considered to be the nutritional micronutrients associated with obesity. Some studies (14–16) have revealed that obesity development rates are higher in places where micronutrient deficiencies are more frequent, while others have discovered that deficiencies in particular micronutrients may be related to increased aberrant fat deposition in the body. The effect of micronutrients on obesity may occur through changing leptin concentrations in the blood, which control food intake and energy expenditure, leading to changes in adipose tissue mass, and also by regulation of the inflammatory response.

However, considering that the impact of micronutrients is usually dependent on their interaction with one another, their overall effect on the body may differ from that of single antioxidant, and their contribution to the development of obesity may change. Therefore, in this study, the 11 antioxidant micronutrients mentioned above were studied as a whole to investigate their relationship with obesity, and then each antioxidant micronutrient was assessed individually to determine its proportional association with obesity, to examine the relationship between each antioxidant micronutrient and the prevalence of obesity.

## 2. Materials and methods

### 2.1. Study and population

The study population was obtained from the National Health and Nutrition Examination Survey (NHANES) database, a major research program of the National Center for Health Statistics (NCHS) that analyzes the health and nutritional status of adults and children in the United States using a mix of interviews and physical examinations for disease preventive reasons. Here, we utilized data from the NHANES What We Eat in America (WWEIA), a research run by the US Departments of Agriculture (USDA) and Health and Human Services (DHHS), to acquire dietary recall data on vitamin intake which are available on the NHANES Dietary Data page^1^ in the study population, from eight consecutive cycles (2003–2018) in 2-year intervals.

Those with less than two valid 24-h food recalls were eliminated, as were participants under the age of 18 or pregnant with missing BMI or waist circumference data from 2003 to 2018. 80312 people were initially included in our analyses. Further, we excluded 10,713 participants with missing data on two valid 24-h dietary recalls and 28,578 people under the age of 18 or with missing data on BMI or being pregnant. Finally, a total of 41,021 and 39,947 participants were enrolled in our subsequent analyses for the association between the antioxidant micronutrients with obesity and abdominal obesity, respectively. The National Health Statistics Research Ethics Review Board authorized all procedures for this investigation, and signed informed permission was received from all participants; moreover, none of the authors of this study were involved in the collection or development of the NHANES database.

### 2.2. Dietary intake collection from two 24-h diet recalls

NHANES has performed two 24-h recalls to mobile examination center (MEC) participants: the first recall is administered in the MEC, and then a second recall is administered by phone 3–10 days later. According to the NHANES database, the dietary intake data are used to estimate the types and amounts of foods and beverages (including all types of water) consumed during the 24-h period before the interview (midnight to midnight), and to estimate intakes of energy, nutrients, and other food components from those foods and beverages.

Excluding nutrients derived through drugs, antacids or dietary supplements, we utilized the R software (Version 4.1.2) to extract the data about intake of 11 antioxidant-related micronutrients from two 24-h diet recalls, including vitamin C[DR1TVC and DR2TVC], iron[DR1TIRON and DR2TIRON], vitamin E as α-tocopherol[DR1TATOC and DR2TATOC], zinc[DR1TZINC and DR2TZINC], β-carotene[DR1TBCAR and DR2TBCAR], copper[DR1TCOPP and DR2TCOPP], α-carotene[DR1TACAR and DR2TACAR], vitamin A[DR1TVARA and DR2TVARA], retinol[DR1TRET and DR2TRET], β-cryptoxanthin[DR1TCRYP and DR2TCRYP], selenium[DR1TSELE and DR2TSELE].^2^ Finally, the average of two days intake, the actual dietary intake instead of the usual or habitual dietary intake, for each antioxidant was used to analyze due to the reason that using the Multiple Source Method (MSM) and the National Cancer Institute (NCI) method, the values are not expressively different when comparing the mean intake estimated using the two-day mean (17).

### 2.3. Obesity definition

Obesity and abdominal obesity were defined that the condition of BMI 30.0 kg/m^2^ are obese, while those who are abdominally obese must meet the condition of waist circumference > 102 cm for men or > 88 cm for women (18).

### 2.4. Covariates

As possible confounders such as socio-demographic traits, lifestyle, and behavioral patterns were identified, the following variables were incorporated into the model. We included factors such as age (years), male (male or female), education level (below, equal to, or above high school), race/ethnicity, and poverty status in the socio-demographics. We also included smoking status, alcohol drinking status, total calorie intake, and sedentary time as life behavioral factors. Moreover, for medical factors, we included HDL-C, total cholesterol, and eGFR, as well as diabetes and hypertension (including SBP and DBP).

### 2.5. Statistical analyses

Eleven antioxidant micronutrients were analyzed in connection to the obese and abdominally obese populations. The normality of continuous variables was tested using the Kolmogorov–smirnov statistic. Continuous variables were reported as mean (standard deviation [SD]) or medians (interquartile ranges [IQRs]) and compared using Student’s t-test (normal distribution) or the Mann–Whitney *U* test (non-normal distribution). After finding that the continuous variable representing antioxidant micronutrients was skewed, we log-transformed it to make it more normally distributed. Alternatively, absolute values (percentages) were used to describe categorical and dichotomous variables, with a *X*^2^ test used for comparison. All antioxidant micronutrients’ correlation coefficients were calculated using the Spearman’s correlation analysis, and the antioxidant micronutrient metabolites were sorted into quartiles with the bottom group serving as a reference.

#### 2.5.1. Statistical model 1: Multivariate logistic regression model

As a preliminary step, odds ratios (ORs) and 95% confidence intervals (CIs) were calculated using multivariate logistic regression models. By comparing the second, third, and fourth quartiles of antioxidant micronutrients with the first quartile, we were able to evaluate the correlation between the 11 micronutrients and the prevalence of adult obesity and abdominal obesity. Three models were also utilized, with constant adjustment for possible confounding variables: model 1, which was not adjusted for by any covariates; model 2, which was based on model 1, adjusted for age, gender, education level, race, and poverty; Model 3, which derived from model 2 with the inclusion of smoker, alcohol user, energy intake, sedentary time, total cholesterol, high-density lipoprotein cholesterol, dietary supplement use, glomerular filtration rate (eGFR), systolic blood pressure (SBP), diastolic blood pressure (DBP), and diabetes as independent variables. We directly normalized total energy intake as a confounding factor in various regression analysis models to adjust the nutrients for the effect of total energy.

#### 2.5.2. Statistical model 2: Weighted quantile sum (WQS) regression model

As a step, to further examine the beneficial correlations of 11 antioxidant micronutrient combinations with the prevalence of obesity and abdominal obesity, we used the WQS regression model, which is a weighted quartile sum technique paired with linear (continuous outcome) or logistic (binary outcome) regression. Additionally, a weighted linear index was computed by modifying the WQS regression model to capture the total body burden of all micronutrients, with individual weights revealing the relative importance of each element to the overall connection.

#### 2.5.3. Statistical model 3: The restricted cubic spline (RCS) regression model

Finally, we utilized log2-transformed concentrations of each micronutrient as continuous variables and BMI or waist circumference as binomial outcome variables to conduct a threshold effects analysis of obesity prevalence. we investigated whether the shape of the relationship between log2-transformed micronutrient intake and the prevalence of obesity and abdominal obesity was non-linear using the restricted cubic spline (RCS) regression model and analysis of variance (ANOVA) plotted at three nodes (10th, 50th, 90th) for antioxidant micronutrients. A segmental linear connection between log2-transformed micronutrient and obesity and abdominal obesity was fitted using segmental regression if the analysis was non-linear, controlling for age, sex, education level, ethnicity, poverty, smoking, alcohol use, calorie intake, sedentary time, total cholesterol, HDL cholesterol, dietary supplement usage, eGFR, SBP, DBP, and diabetes mellitus. Threshold inflection points were recalculated using a recursive technique and tweaked as needed. We considered a value of *p* 0.05 to be statistically significant (two-sided).

## 3. Results

### 3.1. Study participants characteristics

The characteristics of the study population are shown in Table 1, consisting of 20,394 (49.70%) males and 20,627 (50.30%) females with a mean age of 47.98 ± 18.93 years old. Participants with obesity were typically older (49.26 ± 17.36 vs. 47.24 ± 19.75), more men (55.1% vs. 47.5%), more high-school educated individuals (24.7% vs. 22.6%), more poverty (22.4% vs. 21.5%), higher BMI (35.99 ± 5.88 vs. 24.83 ± 3.18), higher level of total cholesterol (193.67 ± 41.20 vs. 190.98 ± 40.98), and more diabetes (18.80% vs. 8.1%). All the above differences between obesity and non-obesity groups were statistically significant (*p* < 0.05).

**Table 1:** Characteristics of the participants in NHANES 2003–2018.

Variable	Total (*n* = 41,021)	Non-obesity (*n* = 25,934)	Obesity (*n* = 15,087)	*p* value
Age, years^*^	47.98 (18.93)	47.24 (19.75)	49.26 (17.36)	<0.001
Male, %^#^	20,394 (49.7%)	13,626 (52.5%)	6,768 (44.9%)	<0.001
Education level, %^#^				<0.001
Below high school	10,163 (24.8%)	6,320 (24.4%)	3,843 (25.5%)	
High school	9,584 (23.4%)	5,855 (22.6%)	3,729 (24.7%)	
Above high school	21,274 (51.9%)	13,759 (53.1%)	7,515 (49.8%)	
Race/ethnicity, %^#^				<0.001
Mexican American	6,793 (16.6%)	4,040 (15.6%)	2,753 (18.2%)	
Other Hispanic	3,599 (8.8%)	2,262 (8.7%)	1,337 (8.9%)	
Non-Hispanic White	17,641 (43.0%)	11,536 (44.5%)	6,105 (40.5%)	
Non-Hispanic Black	8,976 (21.9%)	4,911 (18.9%)	4,065 (26.9%)	
Other race	4,012 (9.8%)	3,185 (12.3%)	827 (5.5%)	
Poverty, %^#^	8,954 (21.8%)	5,576 (21.5%)	3,378 (22.4%)	0.037
Smoker, %^#^	18,237 (44.5%)	11,627 (44.8%)	6,610 (43.8%)	0.046
Drinking, %^#^	28,956 (70.6%)	18,750 (72.3%)	10,206 (67.6%)	
Body mass index, kg/m^2*^	29.2 (6.9)	24.83 (3.18)	35.99 (5.88)	<0.001
HDL-C, mmol/L^*^	1.37 (0.40)	1.44 (0.42)	1.24 (0.34)	<0.001
Total cholesterol, mg/dL^*^	191.97 (41.08)	190.98 (40.98)	193.67 (41.20)	<0.001
eGFR, ml/min/1.73 m^2*^	94.60 (24.38)	95.41 (24.17)	93.20 (24.67)	<0.001
Energy intake, kcal/day^*^	2,054.05 (874.25)	2,085.10 (894.69)	2,000.68 (835.30)	<0.001
Dietary supplement use, %^#^	19,935 (48.6%)	12,818 (49.4%)	7,117 (47.2%%)	<0.001
Sedentary time, hrs^#^				<0.001
<3 h	6,691 (16.3%)	4,516 (17.4%)	2,175 (14.4%)	
3–6 h	19,318 (47.1%)	12,485 (48.1%)	6,833 (45.3%)	
>6 h	15,012 (36.6%)	8,933 (34.4%)	6,079 (40.3%)	
SBP, mmHg^*^	124.03 (18.83)	122.51 (19.21)	126.64 (17.85)	<0.001
DBP, mmHg^*^	69.78 (13.00)	68.77 (12.72)	71.53 (13.28)	<0.001
Diabetes, %^#^	4,926 (12.0%)	2,092 (8.1%)	2,834 (18.8%)	<0.001

Data are presented as mean (SD) or median [interquartile range], or *n* (%). ^*^Numerical variable; ^#^Categorical variable.

### 3.2. Distribution of and correlation among antioxidant micronutrient levels

As a result, Supplementary Table S1 demonstrates the concentrations and distribution of the 11 antioxidant micronutrients, with vitamin C having the highest median intake (50th = 63.5 mg). Iron (50th = 13.1 mg), zinc (50th = 9.9 mg), vitamin E (50th = 6.9 mg),copper (50th = 1.1 mg), β-carotene (50th = 989.5ug), vitamin A (50th = 494.0ug), retinol (50th = 325.0ug), selenium (50th = 101.3ug) and α-carotene (50th = 73.5ug) then follow, with β-cryptoxanthin (50th = 41.0ug) having the lowest.

Furthermore, we analyzed the correlation among 11 antioxidant micronutrients. Most of the antioxidant micronutrients have a relatively high correlation with the other 10 vitamins, according to correlation analysis (Supplementary Figure S2). We found that the correlations between α-carotene and β-carotene, iron and zinc, and selenium and zinc have *r* value >0.7; copper and vitamin E, vitamin A and retinol, β-carotene and vitamin A, iron and vitamin A, copper and vitamin A, vitamin C and β-cryptoxanthin, selenium and iron, copper and iron, copper and zinc and copper and selenium have *r* value of 0.5–0.7 (all *p* < 0.05).

### 3.3. Associations of antioxidant micronutrients with obesity

The reference category was regarded as the lowest quartile among the 11 antioxidant micronutrients when they were separated into quartiles. Table 2 displays the findings from the logistic regression analysis in different models that were adjusted for the covariates to determine the prevalence rate of obesity linked to antioxidant micronutrients. After adjustment of the selected covariates, we found that vitamin A was significantly and negatively associated with obesity (*p* for trend <0.001). When comparing the fourth quartile with the reference quartile, vitamin A has a lower odd ratio (OR) (0.86, 95%CI: 0.80–0.92). Similar associations between α-carotene (OR = 0.90 [0.84–0.96], *p* for trend = 0.010), β-carotene (OR = 0.91 [0.85–0.97], *p* for trend <0.001), β-cryptoxanthin (OR = 0.93 [0.87–0.99], *p* for trend = 0.001), vitamin C (OR = 0.83 [0.78–0.88], *p* for trend <0.001), iron (OR = 0.78 [0.72–0.85], *p* for trend <0.001,), as well as copper (OR = 0.86 [0.79–0.94], *p* for trend <0.001) and obesity were also observed when comparing the fourth quartile with the reference quartile. Reversely, selenium was significantly and positively associated with obesity (*p* for trend <0.001), having a greater OR (1.38, 95%CI: 1.26–1.50) when the fourth quartile is contrasted with the reference quartile. However, no significant association was found between vitamin E, retinol, and obesity.

**Table 2:** Multiple logistic regression associations of antioxidant micronutrients with the prevalence of obesity in adults.

Micronutrients	Quartile 1	Quartile 2	Quartile 3	Quartile 4	*p* for trend
	OR	OR (95%CI)	OR (95%CI)	OR (95%CI)	
**Vitamin E (mg/day)**					
Model 1	1.00 (Ref.)	0.95 (0.90–1.01)	0.90 (0.85–0.95)	0.83 (0.78–0.88)	<0.001
Model 2	1.00 (Ref.)	0.99 (0.93–1.05)	0.97 (0.91–1.02)	0.93 (0.88–0.98)	0.067
Model 3	1.00 (Ref.)	1.04 (0.98–1.11)	1.03 (0.97–1.10)	1.03 (0.96–1.10)	0.672
**Retinol (μg/day)**					
Model 1	1.00 (Ref.)	0.98 (0.92–1.03)	0.97 (0.92–1.03)	0.87 (0.82–0.92)	<0.001
Model 2	1.00 (Ref.)	0.99 (0.93–1.04)	0.99 (0.94–1.05)	0.93 (0.88–0.99)	0.055
Model 3	1.00 (Ref.)	1.01 (0.95–1.07)	1.01 (0.95–1.07)	0.95 (0.89–1.02)	0.203
**Vitamin A (μg/day)**					
Model 1	1.00 (Ref.)	1.01 (0.95–1.06)	0.93 (0.87–0.98)	0.77 (0.72–0.81)	<0.001
Model 2	1.00 (Ref.)	1.03 (0.97–1.09)	0.96 (0.91–1.02)	0.82 (0.77–0.87)	<0.001
Model 3	1.00 (Ref.)	1.03 (0.97–1.10)	1.00 (0.93–1.06)	0.86 (0.80–0.92)	<0.001
**α-carotene (μg/day)**					
Model 1	1.00 (Ref.)	0.97 (0.92–1.03)	0.97 (0.91–1.02)	0.83 (0.79–0.88)	<0.001
Model 2	1.00 (Ref.)	0.95 (0.90–1.01)	0.94 (0.89–0.99)	0.85 (0.80–0.90)	<0.001
Model 3	1.00 (Ref.)	0.97 (0.91–1.03)	0.97 (0.91–1.03)	0.90 (0.84–0.96)	0.010
**β-carotene (μg/day)**					
Model 1	1.00 (Ref.)	1.01 (0.96–1.07)	0.93 (0.88–0.98)	0.80 (0.76–0.85)	<0.001
Model 2	1.00 (Ref.)	1.03 (0.97–1.09)	0.95 (0.90–1.01)	0.83 (0.78–0.88)	<0.001
Model 3	1.00 (Ref.)	1.05 (0.98–1.11)	1.01 (0.95–1.08)	0.91 (0.85–0.97)	<0.001
**β-cryptoxanthin (μg/day)**					
Model 1	1.00 (Ref.)	1.04 (0.98–1.10)	1.01 (0.96–1.07)	0.89 (0.85–0.95)	<0.001
Model 2	1.00 (Ref.)	1.02 (0.96–1.08)	0.99 (0.93–1.05)	0.87 (0.82–0.92)	<0.001
Model 3	1.00 (Ref.)	1.06 (0.99–1.12)	1.02 (0.96–1.09)	0.93 (0.87–0.99)	0.001
**Vitamin C (mg/day)**					
Model 1	1.00 (Ref.)	0.99 (0.93–1.04)	0.86 (0.81–0.91)	0.77 (0.73–0.81)	<0.001
Model 2	1.00 (Ref.)	0.97 (0.92–1.03)	0.84 (0.79–0.89)	0.76 (0.72–0.81)	<0.001
Model 3	1.00 (Ref.)	1.00 (0.94–1.06)	0.89 (0.83–0.95)	0.83 (0.78–0.88)	<0.001
**Iron (mg/day)**					
Model 1	1.00 (Ref.)	0.90 (0.85–0.95)	0.87 (0.83–0.92)	0.73 (0.69–0.77)	<0.001
Model 2	1.00 (Ref.)	0.95 (0.90–1.01)	0.97 (0.91–1.02)	0.85 (0.80–0.90)	<0.001
Model 3	1.00 (Ref.)	0.92 (0.86–0.98)	0.90 (0.84–0.97)	0.78 (0.72–0.85)	<0.001
**Zinc (mg/day)**					
Model 1	1.00 (Ref.)	0.94 (0.89–0.99)	0.89 (0.84–0.94)	0.84 (0.80–0.89)	<0.001
Model 2	1.00 (Ref.)	1.00 (0.95–1.06)	1.01 (0.95–1.07)	1.02 (0.96–1.09)	0.917
Model 3	1.00 (Ref.)	1.05 (0.98–1.12)	1.07 (0.99–1.15)	1.10 (1.01–1.20)	0.157
**Selenium (μg/day)**					
Model 1	1.00 (Ref.)	0.97 (0.92–1.03)	1.02 (0.96–1.08)	0.90 (0.85–0.95)	<0.001
Model 2	1.00 (Ref.)	1.04 (0.98–1.10)	1.18 (1.11–1.25)	1.14 (1.07–1.22)	<0.001
Model 3	1.00 (Ref.)	1.12 (1.05–1.19)	1.33 (1.24–1.43)	1.38 (1.26–1.50)	<0.001
**Copper (mg/day)**					
Model 1	1.00 (Ref.)	0.93 (0.88–0.99)	0.86 (0.81–0.91)	0.70 (0.66–0.74)	<0.001
Model 2	1.00 (Ref.)	0.98 (0.93–1.04)	0.96 (0.90–1.01)	0.83 (0.78–0.88)	<0.001
Model 3	1.00 (Ref.)	0.98 (0.92–1.05)	0.98 (0.91–1.05)	0.86 (0.79–0.94)	<0.001

Model 1 was not adjusted by any covariate; Model 2 was adjusted for age, sex, education level, race, and poverty; Model 3 was adjusted as model 2 plus smoker, alcohol user, energy intake, sedentary time, total cholesterol, high-density lipoprotein cholesterol, dietary supplement use, eGFR, SBP, DBP, and diabetes. OR, Odd ratio; CI, confidence interval; Ref., reference.

**Figure 1:**
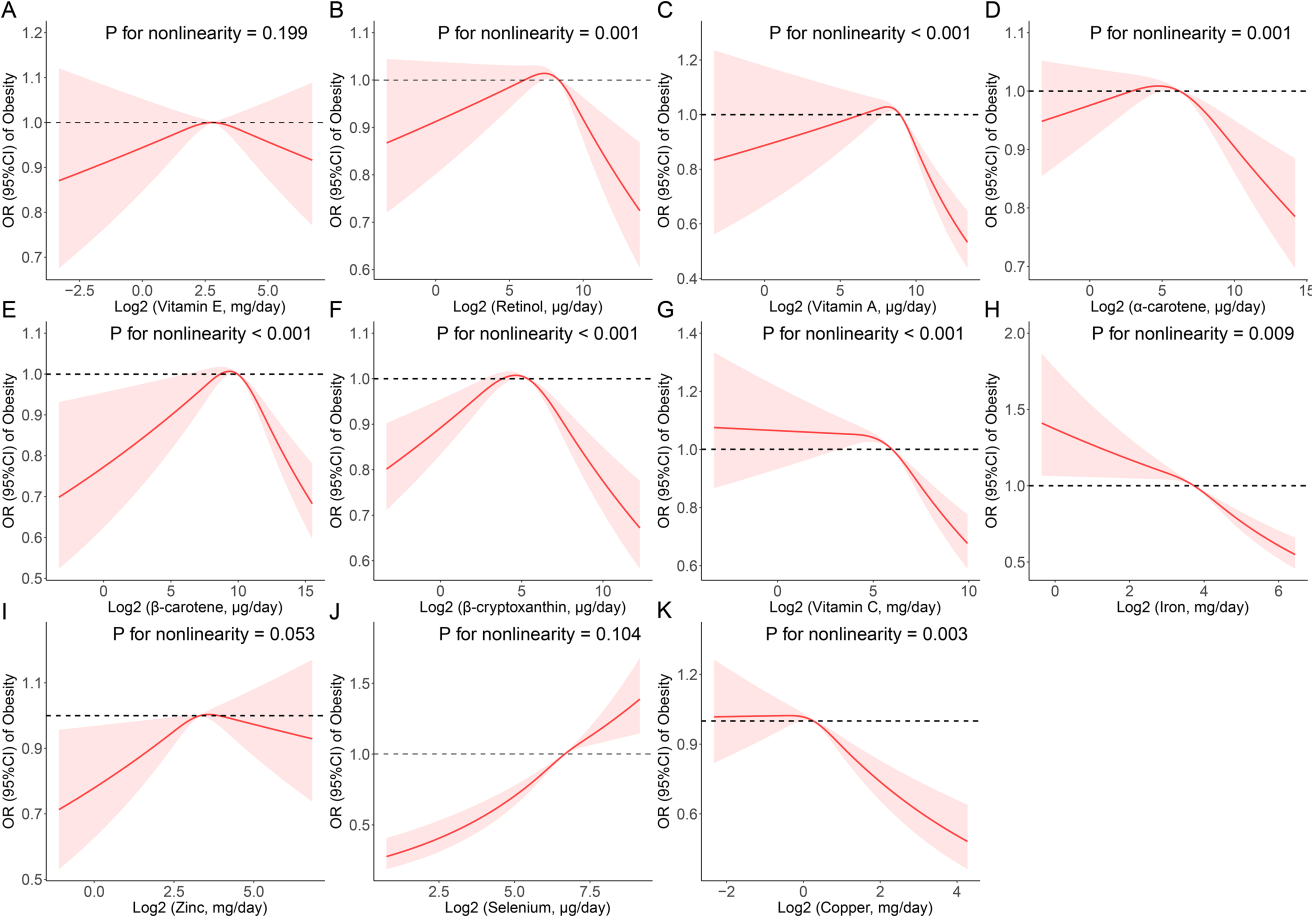
Restricted cubic spline (RCS) analysis with multivariate-adjusted associations between dietary antioxidant micronutrients (**A**: Vitamin E; **B**: Retinol; **C**: Vitamin A; **D**: α-Carotene; **E**: β-Carotene; **F**: β-Cryptoxanthin; **G**: Vitamin C; **H**: Iron; **I**: Zinc; **J**: Selenium; and **K**: Copper) and the prevalence of obesity in adults. Models are adjusted for age, sex, education level, race, poverty, smoker, alcohol user, energy intake, sedentary time, total cholesterol, high-density lipoprotein cholesterol, dietary supplement use, eGFR, SBP, DBP, and diabetes.

According to the results from the restricted cubic spline regression, retinol (*p* for nonlinearity = 0.001), vitamin A (*p* for nonlinearity <0.001), α-carotene (*p* for nonlinearity = 0.001), β-carotene (*p* for nonlinearity <0.001), β-cryptoxanthin (*p* for nonlinearity <0.001), vitamin C (*p* for nonlinearity <0.001), iron (*p* for nonlinearity = 0.009) and cooper (*p* for nonlinearity = 0.003) all had a non-linear association with obesity (Figure 1). Threshold effect analysis demonstrated that the inflection points of retinol, vitamin A, α-carotene, β-carotene, β-cryptoxanthin, vitamin C, iron, and cooper were 235.57, 374.81, 58.89, 891.44, 30.70, 43,410.00, 11,240.00, and 990.00 μg/day, respectively (Supplementary Table S2).Take vitamin C and β-cryptoxanthin for example, when the daily intake of dietary vitamin C exceeded 43.41 mg, the risk of obesity decreased with the increase in daily intake (*p* < 0.05), but when the daily intake was less than 43.41 mg, the trend of increasing risk of obesity gradually stabilized and the results were not statistically significant (*p* > 0.05); while when the daily intake of dietary β-cryptoxanthin exceeded 30.70 ug, the risk of obesity decreased with the increase of daily intake (*p* < 0.05). Similarly, when the daily dietary intake of β-cryptoxanthin was more than 30.70 ug, the prevalence risk of obesity decreased with increasing daily intake (*p* < 0.05), and conversely, when its daily dietary intake was less than 30.70 ug, the prevalence risk of obesity increased with decreasing daily intake (*p* < 0.05).

### 3.4. Associations of antioxidant micronutrients with abdominal obesity

Similar results from logistic regression analysis were also observed between the 11 antioxidant micronutrients and abdominal obesity (Table 3). In the fully adjusted models, compared with the lowest quartile, the ORs of abdominal obesity in the highest quartile were 0.87 (0.81–0.93), 0.87 (0.81–0.93), 0.84 (0.78–0.90), 0.87 (0.81–0.93), 0.79 (0.74–0.85), 0.82 (0.75–0.89), 0.86 (0.79–0.94), and 1.16 (1.06–1.27) for vitamin A, α-carotene, β-carotene, β-cryptoxanthin, vitamin C, iron, copper, and selenium, respectively. Vitamin E, retinol, and zinc were not significantly associated with abdominal obesity, either.

**Table 3:** Multiple logistic regression associations of antioxidant micronutrients with the prevalence of abdominal obesity in adults.

Micronutrients	Quartile 1	Quartile 2	Quartile 3	Quartile 4	*p* for trend
	OR	OR (95%CI)	OR (95%CI)	OR (95%CI)	
**Vitamin E (mg/day)**					
Model 1	1.00 (Ref.)	0.92 (0.87–0.97)	0.85 (0.80–0.90)	0.69 (0.65–0.73)	<0.001
Model 2	1.00 (Ref.)	1.00 (0.94–1.06)	1.01 (0.95–1.07)	0.93 (0.88–0.99)	0.038
Model 3	1.00 (Ref.)	1.05 (0.98–1.12)	1.09 (1.02–1.17)	1.06 (0.98–1.14)	0.098
**Retinol (μg/day)**					
Model 1	1.00 (Ref.)	1.02 (0.97–1.08)	1.06 (1.01–1.12)	0.87 (0.82–0.92)	<0.001
Model 2	1.00 (Ref.)	0.98 (0.92–1.04)	1.02 (0.96–1.08)	0.96 (0.91–1.02)	0.264
Model 3	1.00 (Ref.)	1.01 (0.95–1.08)	1.05 (0.99–1.13)	1.00 (0.94–1.08)	0.355
**Vitamin A (μg/day)**					
Model 1	1.00 (Ref.)	1.10 (1.04–1.16)	1.02 (0.96–1.07)	0.83 (0.79–0.88)	<0.001
Model 2	1.00 (Ref.)	1.06 (0.99–1.13)	0.96 (0.90–1.02)	0.82 (0.77–0.87)	<0.001
Model 3	1.00 (Ref.)	1.08 (1.01–1.15)	1.00 (0.94–1.08)	0.87 (0.81–0.93)	<0.001
**α-carotene (μg/day)**					
Model 1	1.00 (Ref.)	1.02 (0.96–1.08)	1.14 (1.08–1.21)	0.98 (0.93–1.03)	<0.001
Model 2	1.00 (Ref.)	0.94 (0.89–0.99)	0.93 (0.87–0.99)	0.81 (0.76–0.86)	<0.001
Model 3	1.00 (Ref.)	0.97 (0.90–1.03)	0.97 (0.91–1.04)	0.87 (0.81–0.93)	<0.001
**β-carotene (μg/day)**					
Model 1	1.00 (Ref.)	1.00 (0.95–1.06)	1.02 (0.96–1.08)	0.90 (0.86–0.96)	<0.001
Model 2	1.00 (Ref.)	0.99 (0.93–1.05)	0.91 (0.86–0.97)	0.76 (0.72–0.81)	<0.001
Model 3	1.00 (Ref.)	1.00 (0.93–1.06)	0.98 (0.91–1.04)	0.84 (0.78–0.90)	<0.001
**β-cryptoxanthin (μg/day)**					
Model 1	1.00 (Ref.)	1.04 (0.99–1.10)	1.06 (1.00–1.12)	0.93 (0.88–0.98)	<0.001
Model 2	1.00 (Ref.)	0.98 (0.92–1.04)	0.94 (0.89–1.00)	0.81 (0.76–0.86)	<0.001
Model 3	1.00 (Ref.)	1.02 (0.96–1.09)	0.98 (0.92–1.05)	0.87 (0.81–0.93)	<0.001
**Vitamin C (mg/day)**					
Model 1	1.00 (Ref.)	1.02 (0.97–1.08)	0.92 (0.87–0.97)	0.73 (0.69–0.78)	<0.001
Model 2	1.00 (Ref.)	0.94 (0.88–0.99)	0.82 (0.77–0.87)	0.72 (0.68–0.76)	<0.001
Model 3	1.00 (Ref.)	0.97 (0.91–1.04)	0.87 (0.82–0.93)	0.79 (0.74–0.85)	<0.001
**Iron (mg/day)**					
Model 1	1.00 (Ref.)	0.86 (0.82–0.91)	0.75 (0.70–0.79)	0.57 (0.54–0.60)	<0.001
Model 2	1.00 (Ref.)	0.98 (0.92–1.04)	0.97 (0.91–1.03)	0.86 (0.81–0.92)	<0.001
Model 3	1.00 (Ref.)	0.95 (0.88–1.02)	0.92 (0.85–0.99)	0.82 (0.75–0.89)	<0.001
**Zinc (mg/day)**					
Model 1	1.00 (Ref.)	0.87 (0.82–0.92)	0.73 (0.69–0.77)	0.57 (0.54–0.61)	<0.001
Model 2	1.00 (Ref.)	1.00 (0.94–1.06)	0.99 (0.93–1.05)	0.99 (0.93–1.05)	0.960
Model 3	1.00 (Ref.)	1.03 (0.96–1.10)	1.03 (0.95–1.11)	1.04 (0.96–1.14)	0.157
**Selenium (μg/day)**					
Model 1	1.00 (Ref.)	0.85 (0.81–0.90)	0.74 (0.70–0.78)	0.53 (0.50–0.56)	<0.001
Model 2	1.00 (Ref.)	1.01 (0.95–1.07)	1.08 (1.02–1.15)	1.04 (0.98–1.11)	0.062
Model 3	1.00 (Ref.)	1.05 (0.98–1.12)	1.15 (1.07–1.25)	1.16 (1.06–1.27)	0.001
**Copper (mg/day)**					
Model 1	1.00 (Ref.)	0.92 (0.87–0.98)	0.77 (0.73–0.81)	0.58 (0.54–0.61)	<0.001
Model 2	1.00 (Ref.)	1.00 (0.94–1.06)	0.93 (0.88–0.99)	0.82 (0.77–0.87)	<0.001
Model 3	1.00 (Ref.)	1.01 (0.94–1.08)	0.95 (0.88–1.03)	0.86 (0.79–0.94)	<0.001

Model 1 was not adjusted by any covariate; Model 2 was adjusted for age, sex, education level, race, and poverty; Model 3 was adjusted as model 2 plus smoker, alcohol user, energy intake, sedentary time, total cholesterol, high-density lipoprotein cholesterol, dietary supplement use, eGFR, SBP, DBP, and diabetes. OR, Odd ratio; CI, confidence interval; Ref., reference.

**Figure 2:**
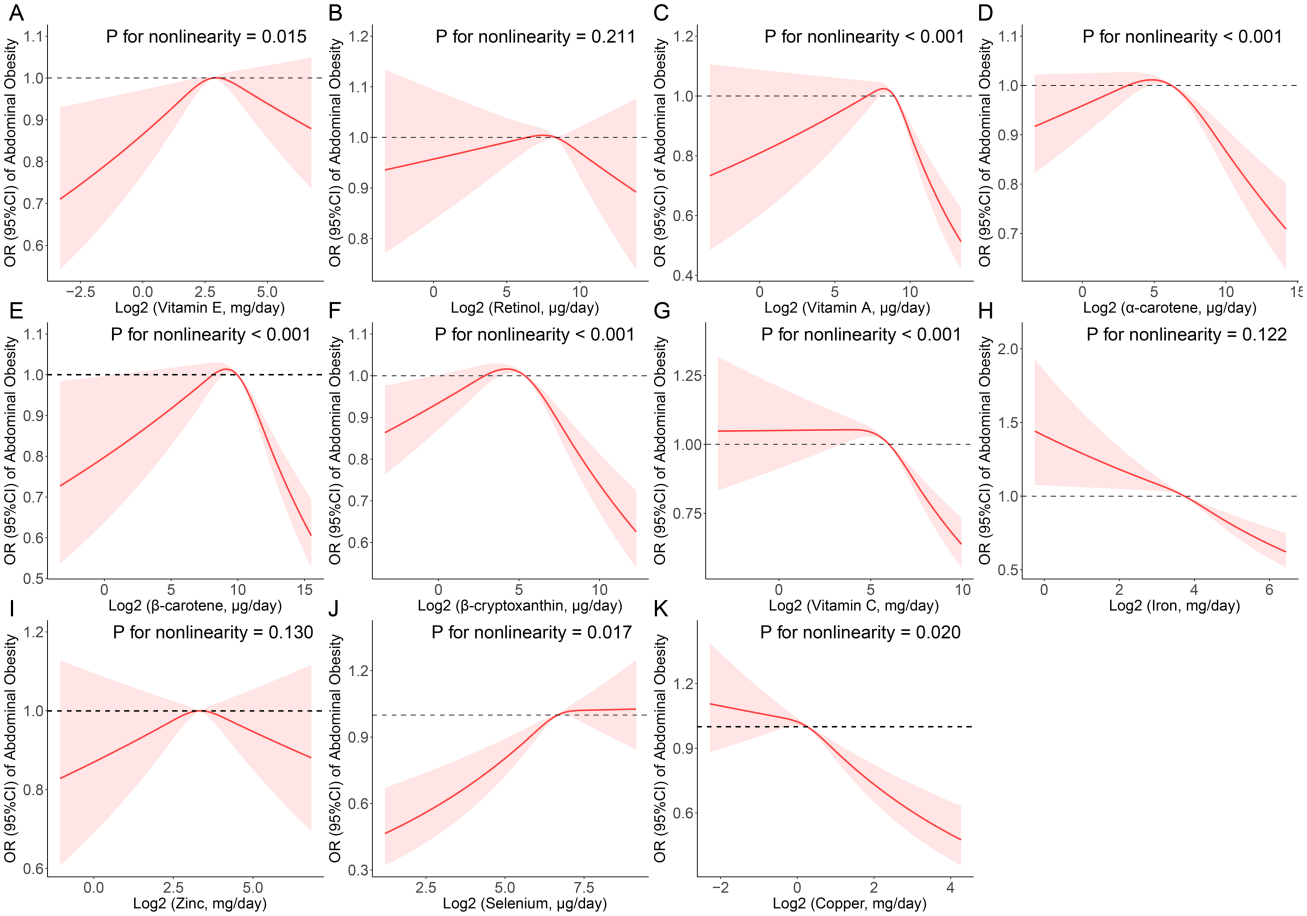
Restricted cubic spline (RCS) analysis with multivariate-adjusted associations between dietary antioxidant micronutrients (**A**: Vitamin E; **B**: Retinol; **C**: Vitamin A; **D**: α-Carotene; **E**: β-Carotene; **F**: β-Cryptoxanthin; **G**: Vitamin C; **H**: Iron; **I**: Zinc; **J**: Selenium; and **K**: Copper) and the prevalence of abdominal obesity in adults. Models are adjusted for age, sex, education level, race, poverty, smoker, alcohol user, energy intake, sedentary time, total cholesterol, high-density lipoprotein cholesterol, dietary supplement use, eGFR, SBP, DBP, and diabetes.

Vitamin E (*p* for nonlinearity = 0.015), vitamin A (*p* for nonlinearity <0.001), α-carotene (*p* for nonlinearity <0.001), β-carotene (*p* for nonlinearity <0.001), β-cryptoxanthin (*p* for nonlinearity <0.001), vitamin C (*p* for nonlinearity <0.001), selenium (*p* for nonlinearity = 0.017) and cooper (*p* for nonlinearity = 0.020) all had a non-linear association with abdominal obesity (Figure 2). Threshold effect analysis demonstrated that the inflection points of vitamin E, vitamin A, α-carotene, β-carotene, β-cryptoxanthin, vitamin C, selenium, and cooper were 7,360.00, 385.34, 55.72, 820.30, 27.86, 42,813.00, 130.69, and 933.00 μg/day, respectively (Supplementary Table S3).

### 3.5. WQS regression analysis between the antioxidant micronutrients and obesity

**Figure 3:**
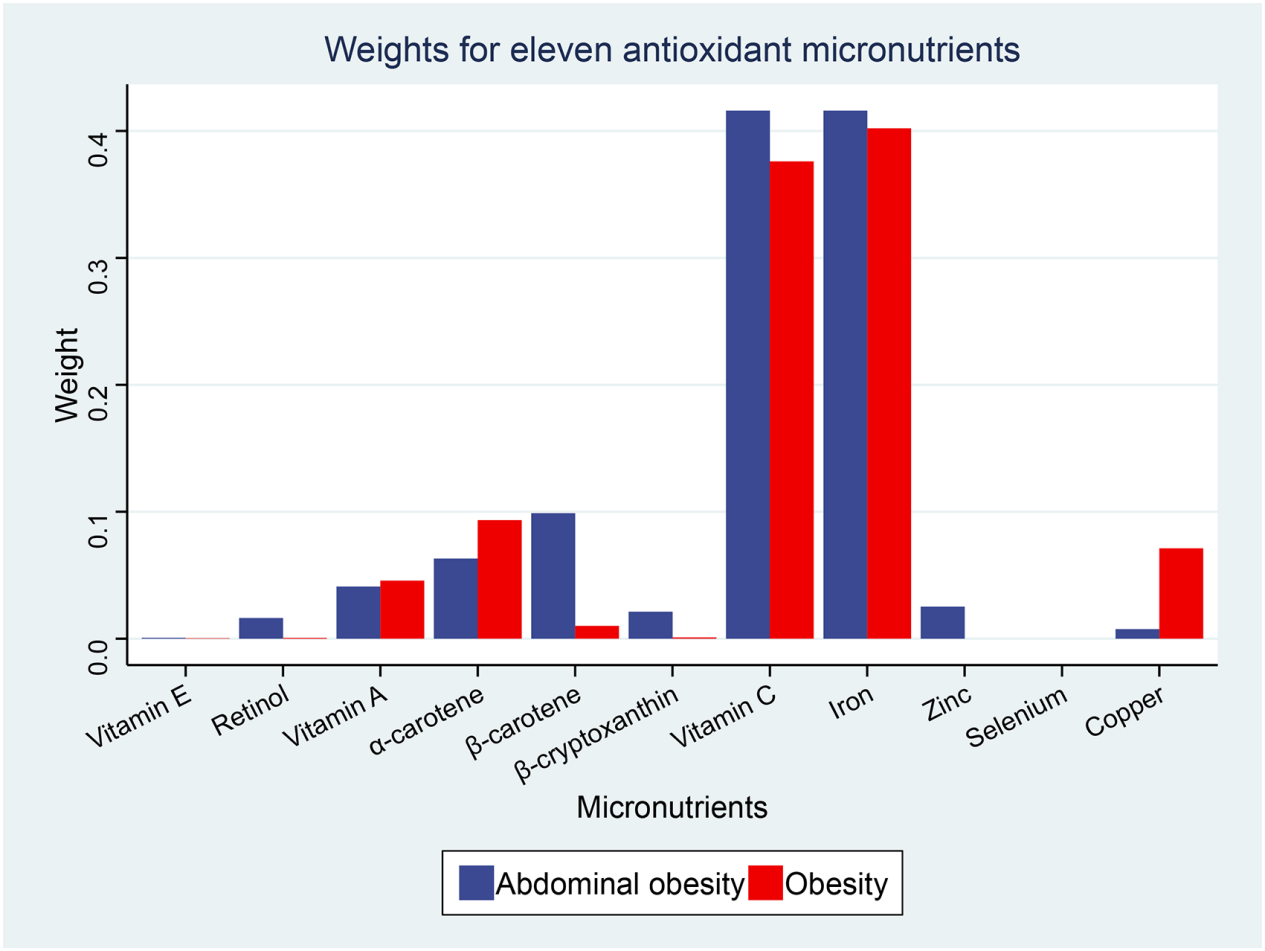
Weights from weighted quantile sum regression (WQS) regression for the mixture of dietary antioxidant micronutrients in relation to the prevalence of obesity and abdominal obesity. Models are adjusted for age, sex, education level, race, poverty, smoker, alcohol user, energy intake, sedentary time, total cholesterol, high-density lipoprotein cholesterol, dietary supplement use, eGFR, SBP, DBP, and diabetes.

Using the WQS regression analysis to explore the inverse associations of the combination of antioxidant micronutrients and obesity. WQS regression limits the direction of exposure-outcome relationships to the negative. The results showed that the combined index for the 11 antioxidant micronutrients was inversely correlated to obesity (adjusted OR = 0.88 [0.84–0.92], *p* < 0.001), abdominal obesity (adjusted OR = 0.87 [0.83–0.91], *p* < 0.001, Table 4). In the negative associations between antioxidant complex and obesity, we found that iron (weight = 40.20%) and vitamin C (weight = 37.60%) had the greatest weight (Figure 3). For the abdominal obesity, iron (weight = 41.60%) and vitamin C (weight = 41.60%) also had the highest weight among the 11 antioxidant micronutrients (Figure 3).

**Table 4:** WQS regression model to assess the protective association of the mixture of eleven antioxidant micronutrients with the prevalence of obesity and abdominal obesity.

	OR	95%CI	*p* value
Obesity	0.88	0.84–0.92	<0.001
Abdominal obesity	0.87	0.83–0.91	<0.001

OR, odds ratio; CI, confidence interval; WQS, weighted quantile sum; WQS regression model was adjusted as age, sex, race, education levels, poverty, smoker, alcohol user, energy intake, sedentary time, total cholesterol, high-density lipoprotein cholesterol, dietary supplement use, eGFR, SBP, DBP, and diabetes.

## 4. Discussion

After correcting for common confounders, our research of 11 dietary antioxidant micronutrients in a cross-sectional, nationally representative general US population revealed that a combination of 11 antioxidant micronutrients was protective against the prevalence of both obesity and abdominal obesity, i.e., a negative correlation existed between obesity and the combination of 11 antioxidant micronutrients. However, we observed that not all antioxidant micronutrients were reliably protective for those who were both overweight and had excess fat in their abdominal regions. In the WQS model, the weight of each micronutrient showed that practically all antioxidants, except selenium, were negatively related to obesity, notably iron and vitamin C. In our multiple logistic regression study, we found that almost all of the antioxidant micronutrients were independently correlated negatively with obesity, except vitamin E, retinol, and zinc, and that selenium was correlated favorably with obesity. Additionally, the RCS analysis showed that there was a nonlinear relationship between obesity and Retinol, vitamin A, α-carotene, β-carotene, β-cryptoxanthin, vitamin C, Iron, and Cooper, with inflection points at 235.57, 374.81, 58.89, 891.44, 30.70, 43,410.00, 11,240.00, and 990.00 g/day, respectively. While inflection points were found at 7,360.00, 385.34, 55.72, 820.30, 27.86, 42,813.00, 130.69, and 933.00 g/day for vitamin E, vitamin A, α-carotene, β-carotene, β-cryptoxanthin, vitamin C, selenium, and Cooper, respectively, in abdominal obesity.

Factors including as genetics, diet, good physical activity, environment, and socioeconomic status all have a role in the development of obesity which is strongly linked to oxidative stress (19). Current evidence shows that oxidative stress may promote obesity by boosting preadipocyte proliferation, accelerating white adipose tissue (WAT) deposition, and modifying food intake and that obesity can produce systemic oxidative stress by numerous mechanisms, including superoxide generation by NADPH oxidase, oxidative phosphorylation, protein kinase C activation, polyol, and hexosamine pathways, further exacerbating obesity (20). In the meanwhile, dietary antioxidants have gained popularity for their ability to neutralize free radicals in the body. As a result, growing studies are looking at the link between fat storage and antioxidant levels in obese people. Aeberli et al. found in Swedish children that dietary intake of antioxidant vitamins (vitamin E and vitamin C and β-carotene) was significantly associated with leptin levels (*p* < 0.05), suggesting that low concentrations of antioxidant vitamins may alter the genetic expression of leptin, leading to the development of leptin resistance and increasing the risk of obesity (21, 22). Another cross-sectional investigation revealed that blood levels of vitamin C were substantially correlated with food intake (23). Vitamin C scavenges free radicals and suppresses lipid peroxidation, which leads to increased vitamin C utilization and decreased vitamin C in the blood in obese people as a result of increased body fat and increased systemic oxidative stress (24). Furthermore, research has revealed that vitamin deficits are linked to abdominal fat formation in obese persons. For example, one Indian research (25) discovered a negative relationship between vitamin C and total body fat. A case–control study in Thailand (26) found a negative association between BMI, waist, and hip circumference and serum concentrations of vitamin E and retinol, and another study (27) showed a significant association between vitamin A and insulin resistance in morbidly obese patients which may be due to the fact that high doses of vitamin A may inhibit the formation of mature adipocytes and also indirectly modifies insulin sensitivity by regulating the production of bioactive proteins secreted by adipocytes, including leptin and resistin. Retinol and carotenoids are precursors to vitamin A. According to certain studies, dietary beta-carotene and its blood levels are favorably associated (28). Serum beta-carotene levels were also shown to be lower in obese persons, most likely because carotenoids are primarily distributed between serum and adipose tissue, and because adipose tissue is an essential storage tissue in humans, a greater proportion of carotenoid intake would be absorbed by adipose tissue in individuals with high adiposity than in those with low adiposity (29–31). This also implies that obese people need consume more carotenoids in order to satisfy their antioxidant requirements. Additionally, research has shown that carotenoids (32–34) which has been proven to improve insulin resistance, reduce the size of adipocytes and body fat tissue, lower pro-inflammatory indicators of obesity such as LDL-c and VLDL-c, and raise high-density lipoprotein cholesterol (HDL-c), among other benefits promote prevention. These previous studies are consistent with our partial results that vitamin A, vitamin C, iron, β-cryptoxanthin, and carotenoids are associated with obesity and are negatively correlated and are an independent protective factor.

Interestingly, we discovered that selenium is not an independent protective factor against obesity, but is positively related to it. Selenium is well-known for working as an antioxidant to influence the thyroid, immune system, and reproduction. Some studies have discovered a significant correlation between dietary selenium intake and obesity. Hawkes et al. study’s revealed that those who consumed more selenium gained weight, whereas those in the low-selenium group lost weight (35). Liver cirrhosis and steatosis, which may be caused by obesity, were shown to be positively correlated with increased dietary selenium intake and blood selenium concentrations by Liu. et al. (36). Furthermore, multiple studies have revealed a link between dietary selenium intake and blood selenium levels and an increased risk of type 2 diabetes in diverse groups (37–39). Meanwhile, Excessive selenium exposure has been seen with similar negative consequences in animal experiments, and one possible explanation is that selenoproteins activate the development of insulin resistance. New evidence suggest that selenium toxicity manifests as oxidative stress, blocked biofilm development, and enzyme function suppression when present in excessive amounts. The results match up with what we found.

Zinc’s possible association with obesity remains controversial. Zinc is a vital vitamin that aids in organism development, reproductive tissue repair, and cellular immunity. It has been linked to the degree of obesity in several studies. Zinc deficiency can stimulate fat deposition and raise body weight (37, 38, 40). Similarly to this, the link between zinc status and obesity may be explained by the interaction between zinc metabolism and leptin, since zinc shortage reduces leptin levels in the blood in humans and rat adipocytes while zinc supplementation has the opposite effect (41, 42).Visceral adipose tissue accumulation was induced by long-term zinc supplementation in mice experiments without the involvement of adipogenesis or adipolysis (43). Based on our findings, we found that zinc does not function as a standalone protective factor against obesity and has a modest protective impact against weight gain. It has been suggested that taking zinc supplements may weaken the immune system, lower HDL cholesterol levels, etc., which could lead to copper deficiency or an increase in copper deficiency (44), but researchers still need to figure out the exact mechanism.

Nevertheless, the antioxidant capacity of nutrients can be influenced by other members of the internal antioxidant network, which is another key factor to consider. For instance, vitamin C and vitamin E are known to work together to renew α-tocopherol in membranes and lipoproteins, which is crucial for protecting protein thiol groups from oxidation (45). The change in carotenoids’ (33, 46) prooxidant and antioxidant behavior has also been found to depend on their interaction with vitamin C and vitamin E in biological membranes. Also zinc status can affect vitamin A metabolism, including its absorption, transport, and utilization (47). Both the zinc-dependent conversion of retinol oxidation to the retina by the action of retinol dehydrogenase and the regulatory function of zinc in vitamin A transport mediated by protein synthesis are believed to be involved in this process.

Therefore, the biological effects of antioxidant micronutrients cannot be adequately explained by a single nutrient model. Recently, researchers in the area of nutritional health have begun to shift their attention from looking at the effects of individual nutrients on health to the effects of being exposed to combinations of nutrients. Here, the inverse association found between obesity and 11 different antioxidant combinations is evaluated using WQS regression, with iron and vitamin C accounting for the bulk of the impact.

Despite being the second most common metal on Earth, iron has poor bioavailability in humans because it often forms very insoluble oxides. Obesity is strongly linked to iron deficiency (48–50), and research (51) has found that one of the pathological mechanisms involved in obesity is iron deposition in the cytoplasm of adipocytes, which results in increased iron content in adipose tissue, possibly as a result of a fourfold increase in intracellular iron content in adipocytes and the expression of iron regulatory proteins. Some animal studies (52, 53) have revealed that a high iron diet may lead to elevated leptin levels in the body, whereas iron produces endocrine dysfunction in adipose tissue and decreases adipocyte lipocalin synthesis. This is consistent with our analysis that iron is independently associated with obesity and vice versa. In addition, we found from a threshold effect analysis that iron intake above 11.24 mg/day may contribute to reduced obesity prevalence. Vitamin C (VC, ascorbic acid) has been shown to prevent obesity by the followings (54): (a) regulating lipid accumulation in adipocytes through direct action on differentiation mechanisms or by modulating motor behavior, (b) inhibiting lipolysis and thus reducing the export of fatty acids to the system, (c) inhibiting glucocorticoid production, and (d) directly interfering with adipose cell-macrophage interactions, (e) scavenging of reactive oxygen species, and (f) possible inhibition of the HIF-1a pathway. Our threshold impact study also revealed that consuming more than 43.41 milligrams of vitamin C per day may have contributed to lower obesity rates. Future dietary strategies for the prevention and treatment of obesity might be devised using these results as a reference dosage, but the precise intake for the population still has to be determined by experimental trials.

Our research has various benefits, not the least of which is that we are the first to explore the connection between 11 micronutrients and obesity in a single cohort. For one, to mitigate mistakes and prevent low correlation components from influencing the analysis, we accounted for the age, education, race/ethnicity, socioeconomic situation as well as other possible complicating variables like (BMI, smoking or not, total energy intake, etc.) which may influence the correlation between antioxidant micronutrients and obesity in the RSC model and the WQS regression model. Second, we have confirmed the accuracy of our findings by verifying them using two distinct models.

However, our study has several limitations. Firstly, we were restricted in our ability to draw causal inferences due to the cross-sectional design of the survey. Second, we may have introduced some unintentional bias into our study by limiting ourselves to focusing on the link between micronutrient intake and obesity and not also considering the role of micronutrient supplementation. At the same time, we did not investigate the relationship between circulating levels of antioxidant micronutrients and obesity, which might be a future research topic. Thirdly, our study’s analysis was limited to the NHANES cohort in the United States; the conclusions would have had greater weight if they had been corroborated by data from other nations or areas.

## 5. Conclusion

According to our research, the combination of 11 antioxidants is negatively associated with the prevalence of obesity, and iron/vitamin C have the greatest weight in the negative associations between antioxidant complex and obesity, as well as abdominal obesity. Future studies are required to analyze and determine the ideal intake amounts to lower obesity in at-risk individuals, as well as to better understand the interactions and complex of many micronutrients and their combined impact on obesity.

## Data availability statement

The original contributions presented in the study are included in the article/Supplementary material, further inquiries can be directed to the corresponding authors.

## Author contributions

YY and HX designed research, drafted the manuscript, performed statistical analysis, extracted the data, and conducted analyses. YZ, LC, and CT took charge of software operation. LM, YC, and BH revised the manuscript. All authors reviewed, edited, and finalized the final version of the manuscript.

## Conflict of interest

The authors declare that the research was conducted in the absence of any commercial or financial relationships that could be construed as a potential conflict of interest.

## Publisher’s note

All claims expressed in this article are solely those of the authors and do not necessarily represent those of their affiliated organizations, or those of the publisher, the editors and the reviewers. Any product that may be evaluated in this article, or claim that may be made by its manufacturer, is not guaranteed or endorsed by the publisher.

## Footnotes

^1^ wwwn.cdc.gov/nchs/nhanes/search/datapage.aspx?Component=Dietary&CycleBeginYear=2018

^2^ https://wwwn.cdc.gov/Nchs/Nhanes/2017-2018/DR1IFF_J.htm#Appendix_5
