# Supplementary material for: Associations of dietary antioxidant micronutrients with the prevalence of obesity in adults
Source: Front Nutr. 2023 Mar 13;10:1098761. doi: 10.3389/fnut.2023.1098761 (PMC10040542; doi:10.3389/fnut.2023.1098761)
Supplement: Supplementary file 1 [file Data_Sheet_1.docx]

**

**

**Supplemental Figure 1.** Eligible participants in the evaluation of the influence between 11 dietary antioxidant micronutrients and the prevalence of obesity in the general adult population.

**Supplement Table 1.** Intake and distribution of the 11 dietary antioxidant micronutrients.

| Micronutrients | N | 5^th^ | 25^th^ | 50^th^ | 75^th^ | 95^th^ |
| --- | --- | --- | --- | --- | --- | --- |
| Vitamin E (mg) | 41021 | 1.9 | 4.3 | 6.9 | 10.8 | 21.8 |
| Retinol (μg) | 41021 | 30.0 | 159.0 | 325.0 | 575.0 | 1202.0 |
| Vitamin A (μg) | 41021 | 116.5 | 298.0 | 494.0 | 764.0 | 1401.0 |
| α-carotene (μg) | 41021 | 1.0 | 22.0 | 73.5 | 404.8 | 1764.4 |
| β-carotene (μg) | 41021 | 110.6 | 400.0 | 989.5 | 2579.3 | 7707.5 |
| β-cryptoxanthin (μg) | 41021 | 1.0 | 13.0 | 41.0 | 111.5 | 391.5 |
| Vitamin C (mg) | 41021 | 7.9 | 29.3 | 63.5 | 116.4 | 234.3 |
| Iron (mg) | 41021 | 5.7 | 9.6 | 13.1 | 18.1 | 28.8 |
| Zinc (mg) | 41021 | 4.1 | 7.1 | 9.9 | 13.7 | 22.1 |
| Selenium (μg) | 41021 | 43.4 | 73.6 | 101.3 | 135.9 | 209.2 |
| Copper (mg) | 41021 | 0.5 | 0.8 | 1.1 | 1.5 | 2.3 |

N, number of participants; 5^th^, 5th percentile; 25^th^, 25th percentile; 50^th^, 50th percentile; 75^th^, 75th percentile; 95^th^, 95th percentile.


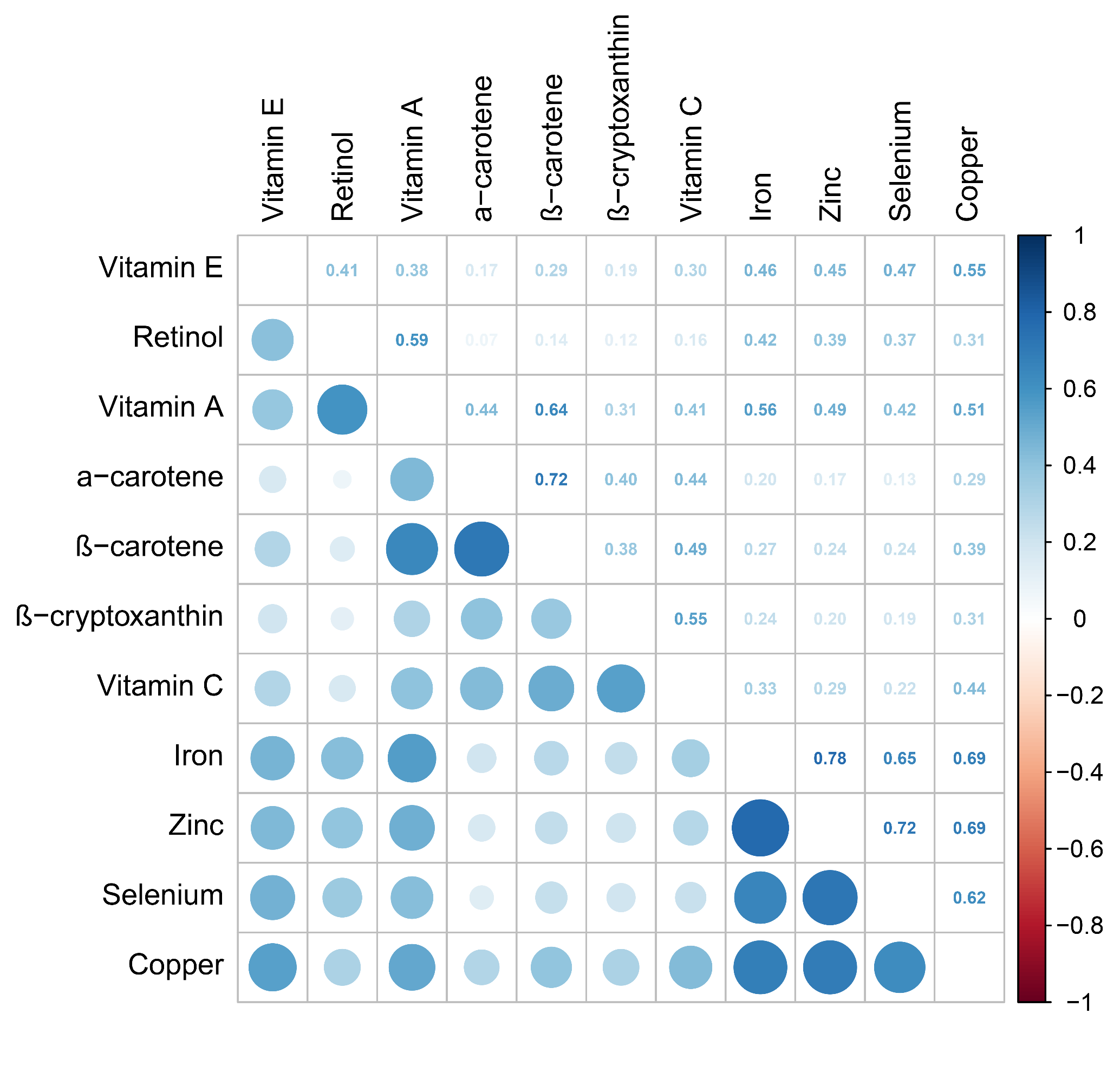


**Supplemental Figure 2.** Pairwise Spearman's correlation coefficients among 11 dietary antioxidant micronutrients in adults.

**Supplement Table 2.** Threshold effect analysis on the prevalence of obesity using piecewise binary logistic regression models.

| Micronutrients | Inflection point | Log2-transformed | Group | OR (95% CI) | *P* value |
| --- | --- | --- | --- | --- | --- |
| Retinol, μg/day | 235.57 | 7.88 | ≤7.88 | 1.01 (0.99-1.03) | 0.277 |
|  |  |  | >7.88 | 0.94 (0.90-0.97) | <0.001 |
| Vitamin A, μg/day | 374.81 | 8.55 | ≤8.55 | 1.02 (0.98-1.06) | 0.823 |
|  |  |  | >8.55 | 0.88 (0.84-0.92) | <0.001 |
| α-carotene, μg/day | 58.89 | 5.88 | ≤5.88 | 1.01 (0.99-1.02) | 0.304 |
|  |  |  | >5.88 | 0.99 (0.97-1.00) | 0.094 |
| β-carotene, μg/day | 891.44 | 9.80 | ≤9.80 | 1.03 (1.00-1.05) | 0.037 |
|  |  |  | >9.80 | 0.94 (0.92-0.97) | <0.001 |
| β-cryptoxanthin, μg/day | 30.70 | 4.94 | ≤4.94 | 1.04 (1.02-1.05) | <0.001 |
|  |  |  | >4.94 | 0.96 (0.94-0.99) | 0.002 |
| Vitamin C, mg/day | 43.41 | 5.44 | ≤5.44 | 1.01 (0.98-1.04) | 0.751 |
|  |  |  | >5.44 | 0.94 (0.90-0.97) | <0.001 |
| Iron, mg/day | 11.24 | 3.49 | ≤3.49 | 1.00 (0.91-1.11) | 0.965 |
|  |  |  | >3.49 | 0.80 (0.74-0.85) | <0.001 |
| Copper, mg/day | 0.99 | -0.02 | ≤-0.02 | 1.09 (0.95-1.24) | 0.235 |
|  |  |  | >-0.02 | 0.83 (0.78-0.89) | <0.001 |

Retinol, Vitamin A, α-carotene, β-carotene, β-cryptoxanthin, Vitamin C, Iron and Copper were log 2 transformed for fitting the piecewise binary logistic regression model. Analyses was adjusted for age, sex, education level, race, poverty, smoker, alcohol user, energy intake, sedentary time, total cholesterol, high-density lipoprotein cholesterol, dietary supplement use, eGFR, SBP, DBP, and diabetes. Abbreviations: OR, Odd ratio; CI, confidence interval.

**Supplement Table 3.** Threshold effect analysis on the prevalence of abdominal obesity using piecewise binary logistic regression models.

| Micronutrients | Inflection point | Log2-transformed | Group | OR (95% CI) | *P* value |
| --- | --- | --- | --- | --- | --- |
| Vitamin E, mg/day | 7.36 | 2.88 | ≤2.88 | 1.04 (0.99-1.09) | 0.133 |
|  |  |  | >2.88 | 0.93 (0.88-0.98) | 0.011 |
| Vitamin A, μg/day | 385.34 | 8.59 | ≤8.59 | 1.02 (0.98-1.06) | 0.419 |
|  |  |  | >8.59 | 0.86 (0.82-0.90) | <0.001 |
| α-carotene, μg/day | 55.72 | 5.80 | ≤5.80 | 1.01 (0.99-1.02) | 0.170 |
|  |  |  | >5.80 | 0.97 (0.95-0.99) | 0.000 |
| β-carotene, μg/day | 820.295 | 9.68 | ≤9.68 | 1.02 (0.99-1.05) | 0.163 |
|  |  |  | >9.68 | 0.92 (0.89-0.95) | <0.001 |
| β-cryptoxanthin, μg/day | 27.857 | 4.80 | ≤4.80 | 1.03 (1.01-1.05) | 0.004 |
|  |  |  | >4.80 | 0.95 (0.93-0.97) | <0.001 |
| Vitamin C, mg/day | 42.813 | 5.42 | ≤5.42 | 1.02 (0.98-1.05) | 0.334 |
|  |  |  | >5.42 | 0.93 (0.90-0.97) | <0.001 |
| Selenium, μg/day | 130.689 | 7.03 | ≤7.03 | 1.14 (1.07-1.22) | <0.001 |
|  |  |  | >7.03 | 0.75 (0.65-0.87) | <0.001 |
| Copper, mg/day | 0.933 | -0.10 | ≤-0.10 | 1.06 (0.90-1.24) | 0.483 |
|  |  |  | >-0.10 | 0.82 (0.76-0.87) | <0.001 |

Vitamin E, Vitamin A, α-carotene, β-carotene, β-cryptoxanthin, Vitamin C, Selenium and Copper were log 2 transformed for fitting the piecewise binary logistic regression model. Analyses was adjusted for age, sex, education level, race, poverty, smoker, alcohol user, energy intake, sedentary time, total cholesterol, high-density lipoprotein cholesterol, dietary supplement use, eGFR, SBP, DBP, and diabetes. Abbreviations: OR, Odd ratio; CI, confidence interval.
